# Analysis of the efficacy of biofeedback for faecal incontinence after surgery for anorectal malformation

**DOI:** 10.1080/07853890.2022.2114607

**Published:** 2022-08-29

**Authors:** Zhenqiang Zhang, Yuan Cheng, Junjun Ju, Weichen Shen, Zhubin Pan, Yuliang Zhou

**Affiliations:** Department of General Surgery, Anhui Provincial Children's Hospital, Hefei, China

**Keywords:** Biofeedback, faecal incontinence, anorectal malformation, children

## Abstract

**Objective:**

To explore the therapeutic effects of biofeedback in the treatment of faecal incontinence (FI) after surgery for anorectal malformation (ARM).

**Methods:**

Clinical data were collected from paediatric patients for postoperative biofeedback due to FI caused by ARM between May 2017 and November 2021. The data included the duration of symptoms, the integrity of the anal sphincter, anorectal manometry parameters, and FI scores. These patients were divided into the low ARM group (group A) and the high ARM group (group B).

**Results:**

A total of 45 paediatric patients were enrolled in the study. There were 28 cases in group A and 17 cases in group B. The differences in age, gender, and body weight were not statistically significant between the two groups (*p* > 0.05). The differences in the clinical indicators were also not statistically significant between the two groups at the time of the initial evaluation. The duration of symptoms was 2.21 ± 0.71 years and 4.14 ± 1.89 years in groups A and B. There were 16 cases with an intact anal sphincter in group A and only two cases with an intact anal sphincter in group B. This difference was statistically significant between the two groups (*p* < .05). The anal resting pressure, initial sensitivity threshold, defaecation sensitivity threshold, defaecation urge threshold, and FI scores were significantly improved in both groups post-treatment compared to pre-treatment (*p* < .001). Strong impulses improved significantly in group A, while strong impulses did not improve significantly in group B. The multivariate logistic regression analysis with these variables further showed that symptom duration and anal sphincter integrity were the main factors influencing the therapeutic effects of biofeedback.

**Conclusion:**

Biofeedback plays a positive role in the treatment of FI in paediatric patients following surgery for ARM. Symptom duration and anal sphincter integrity were found to be the main factors influencing the therapeutic effect of biofeedback.

## Introduction

Biofeedback is currently recommended as a first-line treatment following the failure of conservative treatment methods for defaecation disorders after surgery for anorectal malformation (ARM) [1]. Many studies have been conducted on biofeedback as a treatment for faecal incontinence (FI), with an overall success rate of approximately 75%–80% [2]. Typically, paediatric patients with ARM have good surgical outcomes, but long-term follow-up indicates that approximately 8% of patients will have persistent problems with constipation, FI, and other defaecation disorders [3]. The cause of these symptoms may include anal stenosis, residual segments without nerves, enteric neuronal dysplasia, or rectal malposition [4]. At present, biofeedback is an effective choice for the treatment of defaecation disorders following surgery for ARM. Biofeedback is safer than laxatives, antidiarrheals, botulinum toxin or dextran injections, sacral nerve stimulation, and surgical options. However, the clinical efficacy of biofeedback in paediatric patients remains unknown, and there is a lack of evaluation of the techniques adopted and the outcome of biofeedback in the treatment of defaecation disorders in children. A biofeedback study was launched in our hospital in 2017, and a total of 45 paediatric patients with defaecation disorders following surgery for ARM were treated with pelvic floor biofeedback from May 2017 to December 2021. The present study analyses the therapeutic effects of biofeedback in these patients.

## Materials and methods

### Clinical materials

The study subjects were 45 paediatric patients who underwent pelvic floor biofeedback due to postoperative FI after surgery for anal atresia from May 2017 to December 2021. All patients were operated in our hospital. The inclusion criteria for the study were as follows: (1) patients aged 3–14 years and (2) patients with a definite diagnosis of anal atresia based on preoperative imaging who had been treated with radical surgery. The exclusion criteria for the study were as follows: (1) patients unwilling to participate, (2) patients with anorectal malformations with traumatic sphincter injury or spinal disorders. The enrolled patients were divided into two groups according to the Wingspread classification of ARM: group A was the low and middle ARM group, while group B was the high ARM group. Informed consent to participate was obtained from the families of the paediatric patients, and the study was approved by the ethics committee of Anhui Children's Hospital (EYLL-2022-012). Patients were followed up for a minimum of 1 year and a maximum of 3 years after completion of treatment.

### FI assessment form

St Mark’s incontinence score was adopted to evaluate the severity of FI, as shown in Table 1 [5].

**Table 1.** The assessment form of St. Mark's incontinence score.

	Never	Seldom	Sometimes	Weekly	Daily
Anal incontinence (solid)	0	1	2	3	4
Anal incontinence (liquid)	0	1	2	3	4
Anal incontinence (gas)	0	1	2	3	4
The impact on daily life	0	1	2	3	4
				No	Yes
The need to wear a pad or plug				0	2
The use of constipating medication				0	2
The lack of ability to defer defaecation for 15 min.				0	2

Note: Never: Never happened within the last four weeks; Seldom: With one occurrence within the last four weeks; Sometimes: With more than one occurrence within the last four weeks; Weekly: Occurred on average once or more per week but on average less than once per day during the last four weeks; Daily: Occurred on average one or more times per day for the last four weeks. The minimum score was 0 point with no faecal incontinence. The maximum score was 24 points with total faecal incontinence.

### Pelvic floor biofeedback methods

A bioelectric feedback stimulator manufactured by Laborie, Canada was used to conduct neuromyographic stimulation in paediatric patients with FI following surgery for ARM. One therapeutic cycle consisted of 15 min of neuro-EMG stimulation, followed by 15 min of Kegel training. Patient received one therapeutic cycle daily. Ten therapeutic cycles were one session, with a total duration of 6 months. The current intensity was within the range of 0–20 mA, and the intensity gradually increased from 0 mA to ensure that the paediatric patient could sense the stimulation but did not experience pain. The CONMED electrodes were used. The patch electrodes were placed on the anal lithotomy positions at 3 o'clock, 9 o'clock, and anterior superior iliac spine. The biofeedback therapy was performed by professionally trained attending physicians with operating licence certificates and ten years of experience in the diagnosis and treatment of anorectal diseases. A total of 6 sessions of biofeedback therapy were performed. For children under the age of 5 with good compliance: 1. Parents were asked to communicate effectively to relieve children's resistance; 2. Children were allowed to watch videos of other children undergoing treatment (in compliance with privacy policy); 3. Reward mechanism.

### Anorectal manometry procedures

Anorectal manometry was conducted using an anorectal dynamic analyser produced by Laborie, Canada. In preparation for the manometry, the bowel was cleaned with a glycerine enema (Kaisailu) 2–4 h before the examination. The paediatric patients were then placed in either the left side-lying position or the lithotomy position, and a perfusion catheter with a balloon at the tip was used to detect and record the relevant parameters.

### Statistical methods

Statistical analysis of the clinical data was conducted using SPSS 22.0 software. Measurement data, such as age and body weight, were expressed as mean ± standard deviation or median and inter-quartile ranges, and comparisons between the two groups were conducted using the independent samples t-test (for those that satisfied the normal distribution with homogeneous variance) or the Wilcoxon rank-sum test (for those that did not satisfy the normal distribution or with non-homogeneous variance). The *X^2^* test was adopted for the comparison of countable data, such as gender and medical complications. Fisher’s exact probability test was used when the total number of samples in the fourfold table was less than 40 (*n* < 40) or the frequency less than one (*T* < 1) in at least one of the cells. Binary logistic regression was used to analyse those factors influencing the therapeutic effect, and *p* < .05 was considered statistically significant.

## Results

### Clinical characteristics

The study included 39 males and six females with a male to female ratio of approximately 7:1. The minimum and maximum ages of paediatric patients who underwent biofeedback was three years and 13 years, respectively. In group A, the average age was 8.21 ± 2.92 years. There were two cases with anal atresia combined with atrial septal defects, one case with ventricular septal defects, and one case with a solitary kidney. In group B, the average age was 7.00 ± 6.23 years. There were two cases with anal atresia combined with atrial septal defects, one case with spinal lipoma, one case with thoracic spine deformity, and one case with trisomy 21 syndrome. There was no significant difference in age, gender, and body weight between the two groups (*p* > .05) (Table 2).

**Table 2.** The general characteristics of the clinical data.

Item/Group		Group A	Group B	X^2^ /T	*P*
Age (year)		8.25 ± 2.84	7.00 ± 6.23	1.46	.15
Gender	Male	24	15	0.05	1
	Female	4	2		
Bodyweight (Kg)		22.92 ± 6.23	21.23 ± 6.25	0.88	.38

At the initial assessment, there was no significant difference in the FI scores (12.95 ± 0.4 vs. 13.12 ± 0.42 points), anal resting pressure (27.85 ± 3.45 vs. 27.35 ± 2.57 mmHg), initial sensitivity threshold (92.50 ± 5.69 vs. 90.58 ± 6.34 mmHg), defaecation sensitivity threshold (156.78 ± 7.95 vs. 154.11 ± 8.14 mmHg), and defaecation urge threshold (209.82 ± 6.30 vs. 208.82 ± 7.40 mmHg) between group A and group B. The duration of symptoms (from time of surgery to incontinence) was 2.21 ± 0.71 years and 4.14 ± 1.89 years in groups A and B, respectively. There were 16 cases with an intact anal sphincter in group A and only two cases with an intact anal sphincter in group B. This difference was statistically significant between the two groups (*p* < .05) (Table 3).

**Table 3.** Comparison of the clinical characteristics in the patients between the two groups.

Item/Group		Group A	Group B	X^2^/Z/T	*P*
The age at operation		0.49 ± 0.11	0.47 ± 0.11	0.56	.57
Combined with other deformities	With	9	3	1.137	.286
	Without	19	14		
The duration of symptom (Year)		2.21 ± 0.71	4.14 ± 1.89	−4.03	.01
The anal sphincter integrity	Intact	16	2	9.07	.004
	Not-intact	12	15		
The parameters in anorectal manometry (mmHg)	The anal resting pressure	27.85 ± 3.45	27.35 ± 2.57	0.52	.60
	The initial sensitivity threshold	92.50 ± 5.69	90.58 ± 6.34	1.04	.30
	The defaecation sensitivity threshold	156.78 ± 7.95	154.11 ± 8.14	1.08	.28
	The defaecation urge threshold	209.82 ± 6.30	208.82 ± 7.40	0.48	.63
	The incontinence score	12.95 ± 0.44	13.12 ± 0.42	−1.26	.21

The anal resting pressure, initial sensitivity threshold, defaecation sensitivity threshold, defaecation urge threshold, and FI scores were improved in both groups after treatment compared to pre-treatment (*p* < .001). Strong impulses improved significantly in group A. In group B, the defaecation urge threshold improved following treatment, but the change was not statistically significant (Table 4).

**Table 4.** The assessment of the therapeutic effects.

Item/Group			Before the treatment	After the treatment	T	*P*
Group A	The parameters in anorectal manometry	The anal resting pressure	27.85 ± 3.45	36.25 ± 2.20	−12.28	<.001
		The initial sensitivity threshold	92.50 ± 5.69	138.75 ± 7.53	12.46	<.001
		The defaecation sensitivity threshold	156.78 ± 7.95	135.00 ± 3.04	10.56	<.001
		The defaecation urge threshold	209.82 ± 6.30	215.89 ± 7.82	−3.43	.002
	The faecal incontinence score		12.95 ± 0.44	3.42 ± 0.47	63.08	<.001
Group B	The parameters in anorectal manometry	The anal resting pressure	27.35 ± 2.57	31.23 ± 4.14	−4.22	.001
		The initial sensitivity threshold	90.58 ± 6.34	135.00 ± 9.35	−21.65	<.001
		The defaecation sensitivity threshold	154.11 ± 8.14	139.70 ± 3.13	6.73	<.001
		The defaecation urge threshold	208.82 ± 7.40	209.70 ± 7.99	−0.34	.73
	The faecal incontinence score		13.12 ± 0.42	7.29 ± 0.84	7.80	<.001

The anal sphincter integrity was the influencing factor in the therapeutic effects (Table 6).

Lastly, multiple variables were introduced into the univariate logistic regression analysis. The results confirmed that age, symptom duration, and anal sphincter integrity were the factors influencing the therapeutic outcomes (Table 5). The multivariate logistic regression analysis with these variables further showed that symptom duration and anal sphincter integrity were the main factors influencing the therapeutic effects of biofeedback.

**Table 5.** The univariate logistic regression analysis.

The influencing factor	β	SE	Walds χ2	OR	*P*	95% CI
Age	−0.15	0.11	1.89	0.85	.16	0.68–1.06
Gender	−0.22	0.92	0.58	0.80	.81	0.13–4.91
Bodyweight	−0.46	0.05	0.78	0.95	.37	0.86–1.05
Age at operation	−1.57	2.72	0.33	0.20	.56	0.001–43.23
Comorbidity	−0.79	0.75	1.10	0.45	.29	0.10–1.98
The duration of symptom	1.34	0.43	9.63	3.83	.002	1.64–8.95
The anal sphincter integrity	2.30	0.84	7.44	10.00	.006	1.91–52.29

**Table 6.** The multivariate logistic regression analysis.

The influencing factor	β	SE	Walds χ2	OR	*P*	95% CI
The anal sphincter integrity	1.91	0.98	3.81	6.79	.05	0.99–46.45
The duration of symptom	1.22	0.43	7.77	3.34	.004	1.46–7.90

## Discussion

### Biofeedback therapy

ARM is a relatively common structural deformity in children, with an incidence of approximately 1 in 2,500 live births. Surgical intervention is an important part of the overall chain of treatment for ARM, but it is not the endpoint. For those who have defaecation dysfunction, such as FI and constipation, following surgery for ARM, an objective assessment of the anal function is required, and targeted defaecation training should be actively conducted [6]. With the development of modern medical technology, biofeedback has become widely accepted by the public in recent years, and this can help to avoid invasive secondary surgical treatment for paediatric patients [7]. One study even recommended that following anal surgery, all patients should receive pelvic floor muscle training prior to discharge [8]. Yet, the clinical efficacy of biofeedback in children remains unclear, and there is a lack of objective assessment of the biofeedback techniques used and their outcomes. Available national and international studies suggest that biofeedback has a positive effect on children with FI. In studies conducted by the American Neurogastroenterology and Motility Society and the European Neurogastroenterology and Motility Society, biofeedback is recommended for the short- and long-term treatment of children with defaecation disorders, such as FI and constipation [9]. Similarly, the clinical practice guidelines issued by the American Society of Colon and Rectal Surgeons recommend biofeedback as a treatment modality for constipation and FI [10].

### The mechanism of action of biofeedback

Typically, FI following surgery for ARM is caused by neurological incontinence, which is closely related to changes in rectal sensitivity and anorectal pressure abnormalities. Clinical treatment is based on the principle of promoting the recovery of anal sphincter function and improving anal nerve contraction and defaecation in paediatric patients [11]. In the present study, the paediatric patients were evaluated for FI both before and after treatment using the St. Mark's incontinence score. The therapeutic results found that although approximately one third of these patients still experienced FI following biofeedback, their FI scores improved after treatment, from 12.95 ± 0.44 to 3.42 ± 0.47 points in group A and from 13.12 ± 0.42 to 7.29 ± 0.84 points in group B. These results are similar to those obtained in studies by both van Tets [12] and Vitton et al. [13]. Following the application of biofeedback, the initial sensitivity threshold, defaecation sensitivity threshold, and defaecation urge threshold improved in both groups, with the improvement in anal resting pressure and defaecation urge threshold being the most significant. An earlier study by Lestar et al. investigated the conditions affecting improvement in anal resting pressure and found that 30% of anal resting pressure was due to the tonic activity of the external striated muscle [14]. Therefore, it was speculated that significant improvements in the anal resting pressure and defaecation urge threshold following biofeedback might be due to the direct effect of biofeedback training on the external anal sphincter, and the success of such sensory discrimination training might have a profound effect on therapeutic outcomes. Similar studies have shown that improvements in rectal sensitivity and pressure following biofeedback might be attributed to improvements in the sensory pathways and cortical recognition patterns in the cerebellum [15].

### Influencing factors in biofeedback therapy

Further analysis of the factors affecting therapeutic outcomes in paediatric patients with ARM following surgery found that in a univariate logistic regression, symptom duration and anal sphincter integrity were the principal factors influencing the therapeutic outcome, with the integrity of the anal sphincter being a positive influencing factor. Current studies have shown that in the paediatric population, there are no clearly defined values for the thickness of the external sphincter, and it is controversial whether its morphology and function change with age; however, the degree of anal sphincter injury following anal atresia appears to negatively influence defaecation [16,17]. It is speculated that the shorter the symptom duration, the less atrophy and the higher the integrity of the external anal sphincter and that external sphincter function might be effectively restored with biofeedback. This may be related to the fact that the ability to defaecate autonomously requires adequate innervation of the pelvic floor, rectum, and internal and external anal sphincters [18]. The anatomy and function of these muscles might be disrupted after surgical correction of ARM in paediatric patients, which highlights the importance of carefully preserving the anatomy below the peritoneal reflex site during anorectal surgery and minimising changes in rectal position during reconstruction to ensure minimal loss of function. The above speculation could well explain the results of the multivariate logistic regression analysis, which suggest that anal sphincter integrity is an independent risk factor for biofeedback.

Currently, there are no widely accepted surgical means for postoperative defaecation disorders in paediatric patients with ARM. Commonly adopted methods, such as modified femoral gracilis transfer for the replacement of the external anal sphincter, may achieve autonomous control of faecal discharge through the maintenance of the tight closure of the anal canal by increasing the canal’s anal systolic and resting pressure. This method is used to treat postoperative FI in patients with anal atresia, but its long-term efficacy is not satisfactory [19]. Pelvic floor biofeedback is recommended as an effective treatment for postoperative FI in paediatric patients with ARM [20]. There is, however, a need to better understand the needs of paediatric patients with FI. The number of anorectal doctors specializing in paediatric surgery is small, and many doctors are not aware of the appropriate treatment, leading to great difficulties in the clinical management of paediatric patients with FI. Children who experience FI for a long period can undergo physical and psychological suffering. Therefore, treating these patients appropriately in early childhood and trying to ensure that FI symptoms are controlled before reaching school age can avoid the social consequences associated with FI and may prevent the need for further surgical intervention. Where a paediatric patient does develop social and psychological problems as a result of FI, it is necessary to obtain cooperation from parents, schools, and the wider society to provide the necessary psychological counselling and prompt treatment.

The limitations of this study included poor compliance of most children during the biofeedback therapy, small sample size, short follow-up, and lack of assessment of children’s quality of life.

## Conclusion

The use of biofeedback in paediatric patients to treat FI following surgery for ARM was found to be effective. However, the therapeutic results were found to be influenced by symptom duration and the integrity of the anal sphincter. It is believed that further development of biofeedback technology could help better control and treat postoperative defaecation disorders in paediatric patients with ARM.

## Data Availability

The data that support the findings of this study are available from the corresponding author, [Zhang ZQ], upon reasonable request.
